# Nocturnal oxyhemoglobin desaturation and arteriopathy in a pediatric sickle cell disease cohort

**DOI:** 10.1212/WNL.0000000000004728

**Published:** 2017-12-12

**Authors:** Nomazulu Dlamini, Dawn E. Saunders, Michael Bynevelt, Sara Trompeter, Timothy C. Cox, Romola S. Bucks, Fenella J. Kirkham

**Affiliations:** From Developmental Neurosciences (N.D., F.J.K.), UCL Great Ormond Street Institute of Child Health, London, UK; Hospital for Sick Children (N.D.), Toronto, Canada; Department of Radiology (D.E.S., M.B., T.C.C.), Great Ormond Street Hospital for Children NHS Trust, London, UK; University of Western Australia (M.B., R.S.B.), Perth; and Department of Haematology (S.T.), University College London Hospital, UK.

## Abstract

**Objective:**

The purpose of this study of sickle cell disease (SCD) was to determine whether arteriopathy, measurable as intracranial vessel signal loss on magnetic resonance angiography (MRA), was associated with low nocturnal hemoglobin oxygen saturation (SpO_2_) or hemolytic rate, measurable as reticulocytosis or unconjugated hyperbilirubinemia.

**Methods:**

Ninety-five East London children with SCD without prior stroke had overnight pulse oximetry, of whom 47 (26 boys, 39 hemoglobin SS; mean age 9.1 ± 3.1 years) also had MRA, transcranial Doppler (TCD), steady-state hemoglobin, and reticulocytes within 34 months. Two radiologists blinded to the other data graded arteriopathy on MRA as 0 (none) or as increasing severity grades 1, 2, or 3.

**Results:**

Grades 2 or 3 arteriopathy (n = 24; 2 with abnormal TCD) predicted stroke/TIA compared with grades 0 and 1 (log-rank χ^2^ [1, n = 47] = 8.1, *p* = 0.004). Mean overnight SpO_2_ correlated negatively with reticulocyte percentage (*r* = −0.387; *p* = 0.007). Despite no significant differences across the degrees of arteriopathy in genotype, mean overnight SpO_2_ was higher (*p* < 0.01) in those with grade 0 (97.0% ± 1.6%) than those with grades 2 (93.9 ± 3.7%) or 3 (93.5% ± 3.0%) arteriopathy. Unconjugated bilirubin was not associated but reticulocyte percentage was lower (*p* < 0.001) in those with grade 0 than those with grades 2 and 3 arteriopathy. In multivariable logistic regression, lower mean overnight SpO_2_ (odds ratio 0.50, 95% confidence interval 0.26–0.96; *p* < 0.01) predicted arteriopathy independent of reticulocyte percentage (odds ratio 1.47, 95% confidence interval 1.15–1.87; *p* = 0.003).

**Conclusion:**

Low nocturnal SpO_2_ and reticulocytosis are associated with intracranial arteriopathy in children with SCD. Preventative strategies might reduce stroke risk.

Patients with sickle cell disease (SCD) and stroke typically have stenosis or occlusion of the arteries of the Circle of Willis^1^ detectable with magnetic resonance angiography (MRA).^2^ Cerebral artery vessel turbulence or signal loss on MRA is associated with perfusion abnormality.^3^ There are few MRA data in unselected asymptomatic patients with SCD and risk factors for MRA abnormality remain unclear.^4,5^

Sleep-disordered breathing is a risk factor for stroke^6^ and carotid artery intima-media thickness, wall diameter, and plaque formation in adults^7,8^; the strongest association is with low hemoglobin oxygen saturation (SpO_2_).^9,10^ Episodic nocturnal oxygen desaturation (NOD) is common in children with SCD,^11^ in part related to upper airway obstruction secondary to adenotonsillar hypertrophy. Continuous NOD affects up to 40% of children with SCD^12^ and may persist during the day.^13^ Increased inflammation is associated with both episodic and continuous NOD in SCD.^13,14^ CNS events, including stroke, in untreated patients have been associated with obstructive sleep apnea (OSA)^15^ and lower prestroke daytime and nocturnal SpO_2_.^16,17^ Maximum internal carotid artery (ICA)/middle cerebral artery (MCA) velocities on transcranial Doppler (TCD) are associated with daytime SpO_2_ independently of haemoglobin.^18,19^ In this secondary analysis of cross-sectional data from the East London cohort, we hypothesized that the severity of NOD would be associated with the degree of arteriopathy, measured as turbulence^17^ or signal loss in the intracranial vessels on MRA. Cerebrovascular disease may be associated with hemolysis,^20,21^ so we included steady-state reticulocyte count and unconjugated bilirubin in the statistical models. The data have been published in abstract form.^22^

## METHODS

### Patients.

From January 1, 1991, until December 31, 1993, all children without previous stroke regularly attending the hemoglobinopathy clinic of Queen Elizabeth Hospital, Hackney, were invited to participate in a prospective study designed to examine whether abnormal TCD and overnight pulse oximetry studies predicted CNS events. Asymptomatic children over the age of 7 years were invited to undergo MRI and MRA without sedation. All MRA scans were undertaken in asymptomatic children and as this cohort was recruited before the results of the Stroke Prevention Trial in Sickle Cell Anemia (STOP) trial were available,^23^ children with abnormal TCD were not transfused. Children were followed until they had a recurrent stroke or TIA or, if they did not, until April 30, 2000.

### Standard protocol approvals, registrations, and patient consents.

Approval was granted by the local National Health Service Research Ethics Committee and written informed consent was obtained from the parents of all participants.

### Overnight sleep studies.

Overnight studies were performed using a Biox 3700 pulse oximeter (Datex-Ohmeda; Hatfield, Hertfordshire, UK) to record SpO_2_ during sleep in 63 patients at home and, in the rest of the study population, in the hospital sleep laboratory.^17^ The results were analyzed before the MRI data were available. We recorded the mean and minimum SpO_2_ and the proportion of sleep spent at SpO_2_ less than 90% and less than 80%, and examined the trace for dips (>4% from baseline) in oxygenation associated with acute pulse rate rises. Patients with symptoms of OSA were referred for consideration of adenotonsillectomy.

### Transcranial Doppler studies.

Transcranial Doppler was undertaken routinely in clinic and was classified as standard risk, conditional, or abnormal (maximum ICA/MCA velocity <170 cm/s, 170–199 cm/s, or ≥200 cm/s, respectively).^24^

### Magnetic resonance studies.

Magnetic resonance (MR) studies were performed on a 1.5T S Vision whole body imaging system (Siemens AG; Erlangen, Germany).^3^

#### Structural MRI.

The structural MRI investigation included sagittal and coronal T1-weighted images (repetition time [TR] 570 ms, echo time [TE] 14 ms), axial turbo spin echo T2-weighted images (TR 3,458 ms, TE 96 ms), and coronal turbo fluid-attenuated inversion recovery T2-weighted images (TR 9,999 ms, TE 119 ms, inversion time 2,210 ms).

#### MR angiography.

MRA was performed using a 3D time-of-flight method, acquiring 3 slabs, each of 3.2 mm thickness, centered on the circle of Willis (TR 35 ms, TE 7.2 ms, flip angle 20°). Interobserver variability was compared without discussion by 2 neuroradiologists (M.B. and D.E.S.) in a set of MRAs examined carefully for evidence of arteriopathy, demonstrable as turbulence or signal loss, particularly in the terminal ICA and in the A1, M1, M2, P1, and P2 segments of the basal vessels. Arteriopathy on MRA was graded according to severity of signal loss (0, none; 1, minor signal attenuation; 2, obvious signal attenuation, but presence of distal flow; 3, signal loss and no distal flow; figure) and the worst arteriopathy in any vessel was recorded. In addition, moyamoya collaterals were also noted. For the full cohort, MRAs were reviewed by M.B. and T.C.C. and grading agreed after discussion, but blind to the oximetry data.

**Figure:**
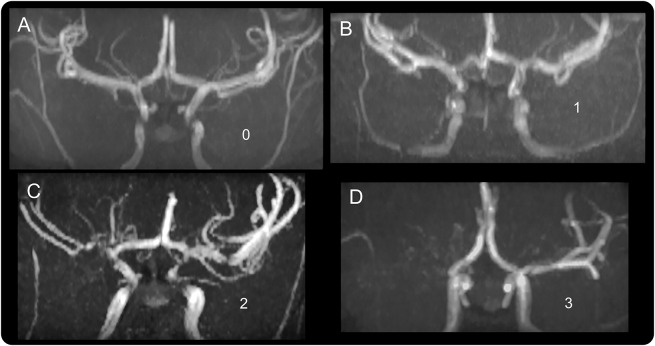
Arteriopathy on magnetic resonance angiography (MRA) in sickle cell disease Arteriopathy on MRA graded as 0, none (A); 1, minor signal attenuation (B); 2, obvious signal attenuation, but presence of distal flow (C); 3, signal loss (D).

### Laboratory investigations.

Laboratory data were available from a cross-sectional study of patients in steady state, i.e., clinically well with no pain or other complications, undertaken in 1994.^25^

### Statistical analysis.

All variables were examined for normality (Shapiro-Wilk) and for those that were not normally distributed, nonparametric descriptives and statistical tests were selected where required. Using Kappa statistics, reproducibility for any degree of arteriopathy was documented in those patients who had repeat MRA within 3 years or at any time rated by T.C.C., M.B., or D.E.S. and interobserver variability was compared in a set of MRAs rated by M.B. and D.E.S. without discussion. Presence of arteriopathy on MRA was compared with TCD categories using χ^2^. Between MRA groups, continuous variables were compared using analysis of variance (ANOVA) or Kruskal-Wallis one-way ANOVA, as appropriate. Kaplan-Meier survival curves and the log-rank test were used to compare TCD or MRA arteriopathy as a predictor of ischemic stroke or TIA. Logistic regression analysis was used to determine the independent contributions of variables to the prediction of normal and mild vs moderate or severe arteriopathy on MRA. Only variables that differed between arteriopathy groups at the univariate level were entered into the multivariable regression model. We assumed statistical significance at *p* < 0.05 but present confidence intervals to clarify the exact strength of statistical relationships. Analysis was conducted using SPSS (release 20.0) (SPSS Inc., Chicago, IL).

## RESULTS

### Patient characteristics.

Data were collected prospectively in 147 children seen before April 2001 for TCD screening, of whom 95 had an overnight pulse oximetry study lasting 4.3–8.2 (median 7.5) hours.^17^ 3D time-of-flight MRA data^3^ were available for 56 of these children a median of 0.5 (interquartile range [IQR] 17.3) months from the sleep study. Forty-eight had HbSS, 4 had HbSβ_0_ thalassemia, and 4 had HbSC disease; 30 (54%) were boys and median age at the time of MR scanning was 9 (IQR 5) years. Forty-seven children (26 boys, 39 with HbSS, 4 with HbSβ_0_ thalassemia, and 4 with HbSC disease; mean age 9.1 ± 3.1 years) had overnight oximetry studies within 3 years of their MRA and form the cohort for this analysis. Four had a stroke and 6 a TIA at a median of 4.12 (range 1.40–6.03) years after MRA.

### Validation of the arteriopathy scale.

Kappa was 0.90 for masked rating of the index MRA using the arteriopathy scale in 14 patients, i.e., excellent interobserver reliability. MRA was repeated at a median of 3.16 (range 0.26–9.38) years in 31 of the children; kappa for comparison of presence of arteriopathy at the 2 time points was 0.36 (fair agreement) but kappa was 1.0 (excellent agreement) for comparison of presence of arteriopathy in the 11 who had a second MRA within 3 years.

### Arteriopathy on MRA and TCD and prediction of stroke or TIA or silent infarction.

Seventeen children had normal MRA, while 6, 11, and 13 had grades 1, 2, and 3 arteriopathy, respectively. None had moyamoya collaterals. The 2 children with abnormal TCD and 1 of the 2 with conditional TCD at baseline had severe arteriopathy on MRA, while the other with conditional TCD at baseline had moderate arteriopathy. The 2 with abnormal TCD both had an ischemic stroke subsequently, as did 2 with normal TCD; although none of the 7 children with conditional TCD at any time had a stroke, one had a TIA. Despite the small number of events in this subset,^17^ survival analysis showed that abnormal TCD at baseline predicted stroke or TIA in the full dataset (log rank χ^2^ [1, n = 47] 6.369; *p* = 0.012) or including only patients with HbSS (log rank χ^2^ [1, n = 39] = 4.845, *p* = 0.028).

All 4 of those who had a stroke at follow-up had moderate or severe arteriopathy on MRA at baseline, as did 5 of 6 of those who had a subsequent TIA. Grade of arteriopathy on MRA predicted stroke or TIA in the full dataset (log rank χ^2^ [3, n = 47] = 12.352, *p* = 0.006) or including only patients with HbSS (log rank χ^2^ [3, n = 39] = 8.233, *p* = 0.041).

Two children had new silent infarction on follow-up MRI, 1 with grade 0 and the other with grade 1 MRA; both had standard risk TCD.

### Associations with arteriopathy on MRA.

There was no difference in the distribution of arteriopathy rankings for male and female participants (χ^2^ [3, n = 47] = 4.55, *p* = 0.208) (in arteriopathy categories 0, 1, 2, 3, there were 11/17, 5/6, 4/11, and 6/13 male participants, respectively), or in the distribution of age across the arteriopathy groups (*F* < 1). One child with HbSβ_0_ thalassemia had grade 2 arteriopathy while the other 7 with compound genotypes had normal MRA and the remaining 29 with arteriopathy had HbSS (χ^2^ [6, n = 47] = 12.26, *p* = 0.056).

There were no differences across the different degrees of arteriopathy in unconjugated bilirubin (*F*_3,40_ = 0.127, *p* = 0.944) or aspartate transaminase (*F*_3,40_ = 0.068, *p* = 0.977). There were, however, differences in hemoglobin (*F*_3,46_ = 3.646, *p* = 0.02) and in reticulocyte percentage (*F*_3,46_ = 12.281, *p* < 0.001). Post hoc analysis revealed lower hemoglobin in grade 3 arteriopathy compared with patients with no vascular disease (mean difference −1.78, *p* = 0.017), and lower mean reticulocyte percentage in patients with no vascular disease compared with those with grade 2 and grade 3 arteriopathy (mean differences, −7.57, *p* = 0.001, and −10.42, *p* < 0.001, respectively) (table 1).

**Table 1:**
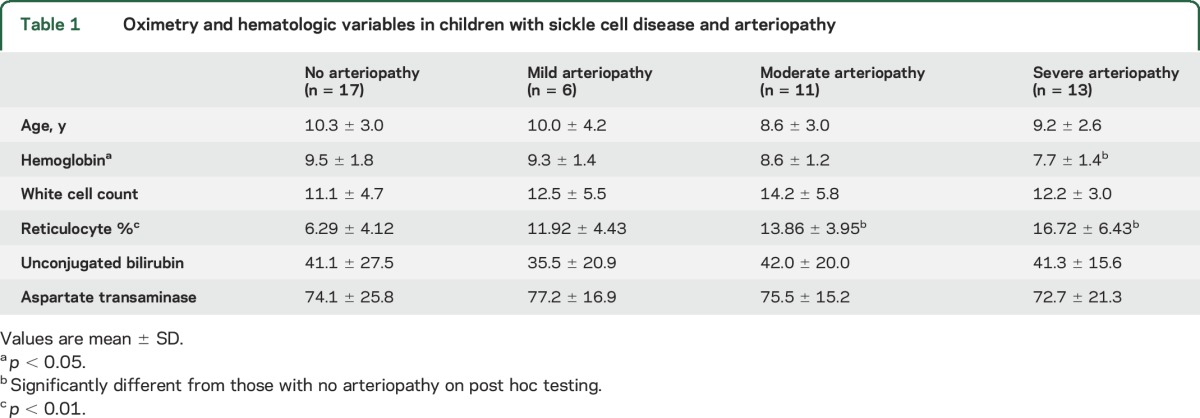
Oximetry and hematologic variables in children with sickle cell disease and arteriopathy

There was also a difference in mean overnight SpO_2_ for those with different degrees of arteriopathy (*F*_3,46_ = 5.11, *p* = 0.004). Post hoc analysis revealed higher mean overnight SpO_2_ in patients with no vascular disease compared with those with grades 2 and 3 arteriopathy (mean difference 3.08%, *p* = 0.024, 3.50%, *p* = 0.005, respectively) (table 1). Repeating this analysis for those with HbSS alone did not change this result (*F*_3,35_ = 3.77, *p* = 0.02); mean difference in mean overnight SpO_2_ in patients with no vascular disease compared with those with grade 2 and 3 arteriopathy was 3.49% and 3.52%, respectively (*p* = 0.046 and 0.028, respectively).

Reticulocyte percentage was higher in those with lower overnight mean oxyhemoglobin saturation (*r*[47] = −0.387, *p* = 0.007). Given the combined reticulocyte and oximetry findings, in 2 separate models, the children were recategorized into 2 arteriopathy groups contrasting (1) no with mild, moderate, or severe arteriopathy and (2) no or mild arteriopathy with moderate or severe arteriopathy for the logistic regression (tables 2 and 3). As there were no differences in the variables significantly associated with arteriopathy in the univariable analysis, multivariable logistic regression was undertaken comparing no with mild, moderate, or severe arteriopathy.

**Table 2:**
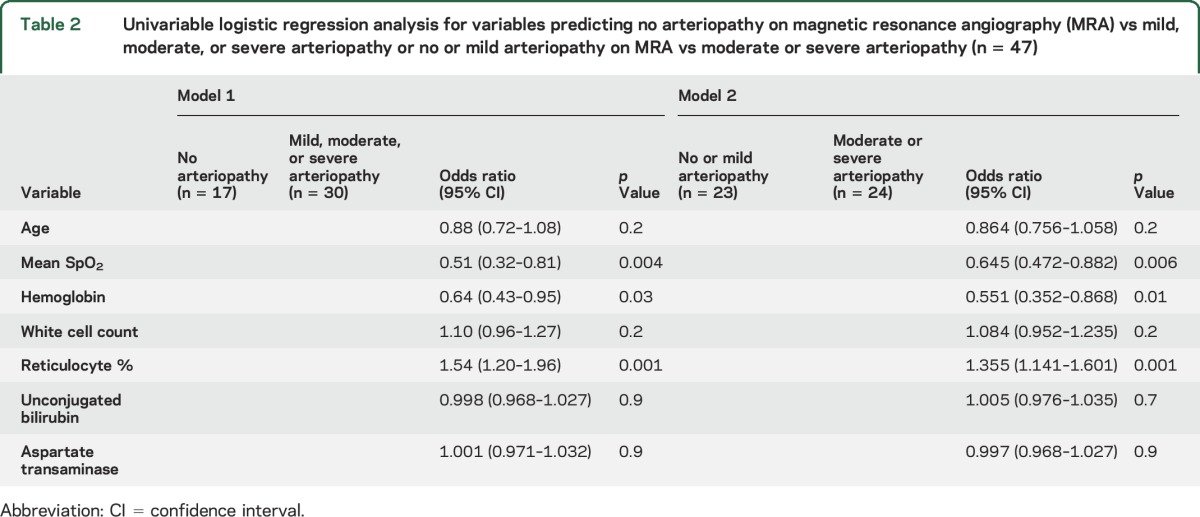
Univariable logistic regression analysis for variables predicting no arteriopathy on magnetic resonance angiography (MRA) vs mild, moderate, or severe arteriopathy or no or mild arteriopathy on MRA vs moderate or severe arteriopathy (n = 47)

**Table 3:**
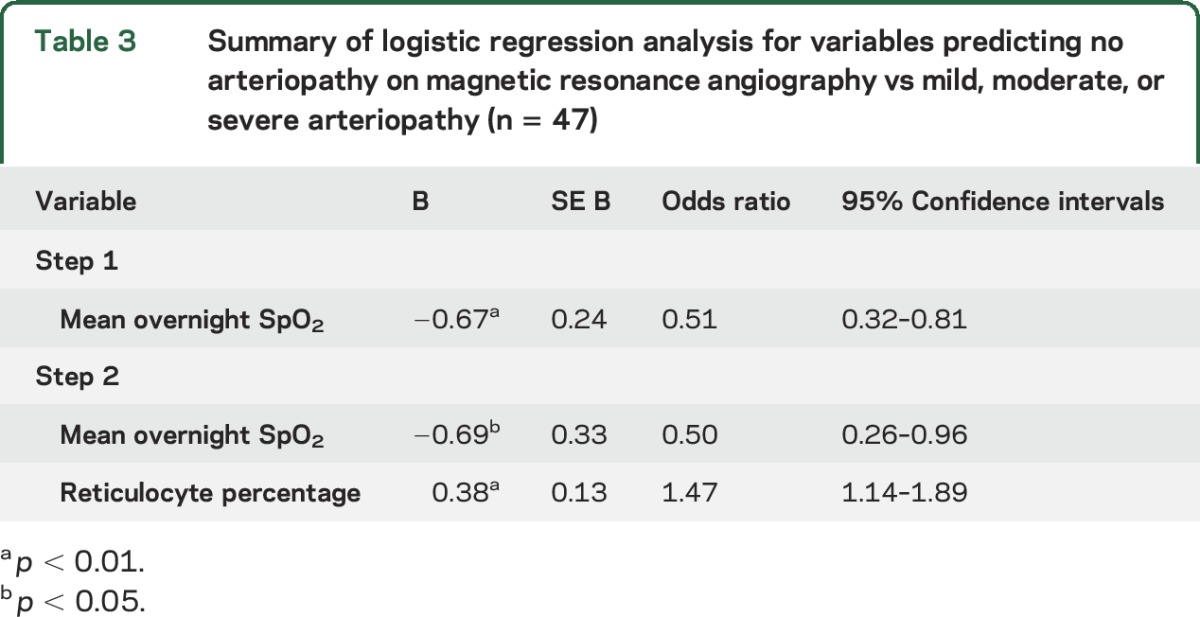
Summary of logistic regression analysis for variables predicting no arteriopathy on magnetic resonance angiography vs mild, moderate, or severe arteriopathy (n = 47)

Logistic analysis was conducted retrospectively to examine the effect of mean overnight hemoglobin saturation (step 1) and reticulocyte percentage (step 2) on no (grade 0; n = 17) vs mild, moderate, or severe (grades 1, 2, or 3; n = 30) arteriopathy. Mean overnight hemoglobin oxygen saturation predicted arteriopathy category (χ^2^ [1, n1 = 17, n2 = 30] = 15.93, *p* < 0.001, *R*^2^ = 0.29, 68.1% correct classification). When reticulocyte percentages were added (step 2), the explanatory power of the model increased (χ^2^ [1, n1 = 17, n2 = 30] = 18.09, *p* < 0.001, *R*^2^ = 0.52, 80.9% correct classification). As can be seen from the summary in table 2, lower mean overnight hemoglobin oxygen saturation and higher reticulocyte percentage were both independent predictors of mild to severe MRA arteriopathy.

## DISCUSSION

There are few data on the relationship among hemoglobin oxygen saturation, markers of hemolysis, and vascular abnormality in SCA.^5,20^ Our data demonstrate that, in unselected initially asymptomatic children with SCD, higher reticulocyte percentage and overnight hemoglobin oxygen saturation are independently associated with the degree of arteriopathy on MRA. The lack of association with unconjugated bilirubin and aspartate transaminase means that it is possible that the association with reticulocytosis may represent erythropoiesis in response to hypoxic exposure rather than hemolytic rate.

Possible mechanisms underlying daytime and nocturnal hemoglobin oxygen desaturation include OSA, chronic lung disease, right-to-left shunting at atrial or pulmonary level with pulmonary hypertension, and abnormality in hemoglobin oxygen affinity.^21^ Our data suggest that the intracranial vessel abnormalities in SCD are also related to low hemoglobin oxygen saturation directly, as well as via a correlation with reticulocytosis. There is some evidence for a link between hypoxia and processes that might lead to nonatherosclerotic vasculopathy, including vasoconstriction,^26^ medial necrosis,^27^ and intimal hyperplasia.^28^

The correlation between reticulocyte percentage and degree of desaturation and the independent effects of reticulocytosis and nocturnal hemoglobin oxygen desaturation on the severity of MRA arteriopathy in our data is intriguing. Reticulocyte percentage is an independent predictor of cerebrovascular disease in children with SCD.^29,30^ Interventions that improve hemoglobin oxygen saturation and reduce the reticulocytosis in sickle cell disease (e.g., transfusions, hydroxycarbamide) may decrease many of the observed vaso-occlusive phenomena.

In our data, the main association with the degree of arteriopathy in the MCAs was low baseline hemoglobin oxygen saturation and there was no association with dips in hemoglobin oxygen saturation or the minimum hemoglobin oxygen saturation. Anemia was also associated with the degree of arteriopathy on MRA, but not as strongly as the hemoglobin oxygen saturation. Interestingly, although there is evidence that chronic inflammation may be associated with the severity of vasculopathy, the white count at the time of the study was not associated with the degree of arteriopathy on MRA in our data. A larger dataset, perhaps incorporating measures of inflammation from earlier in childhood, might be instructive, as MRA abnormality is probably the end stage of a protracted and complex process.

Constriction, stenosis, and occlusion in vessels may be seen as flow-induced signal attenuation on time-of-flight MRA, which we have categorized as degree of signal loss as the structure of the vessel wall is not imaged. It is rarely justifiable to obtain conventional arteriography and pathologic data are likely to remain scarce, but arteriopathy may be documented even in very young children using TCD, allowing study of the effects of exposure to hemoglobin oxygen desaturation and any interaction with hemolysis.

Nocturnal hemoglobin oxygen desaturation as a result of OSA is common in childhood. However, in the absence of SCD, cardiac disease, or an intercurrent illness, arterial ischemic stroke in this group is rare. It is possible that the common link between the pathophysiologic mechanisms of stroke in adulthood and childhood and nocturnal hemoglobin oxygen desaturation is that the first hit of damaged arterial endothelium, perhaps as a result of infection or mechanical and cellular factors in SCD, is followed by the second hit of nocturnal hemoglobin oxygen desaturation with or without sleep-disordered breathing, resulting in medial necrosis or a vicious cycle of intimal hyperplasia and leading to steno-occlusion, which increases the risk of stroke.

We found that the children with abnormal TCD had severe arteriopathy on MRA, in contrast to only 25% of those studied as part of the STOP trial,^31^ and MRA abnormality was also documented in a number of children with standard risk TCD. It might be argued that the long TR used in this study meant that MRA abnormality was overreported but the interobserver reliability and reproducibility for studies repeated in the short and medium terms were excellent. We have previously shown a relationship between MRA abnormality and focal perfusion deficits^3^ and MRA abnormality predicted all the subsequent strokes and all but one of the TIAs, suggesting that the arteriopathy detected was clinically significant. The main weakness is that this was a secondary analysis of data collected for 3 cross-sectional studies undertaken in asymptomatic children with SCD within 3 years. Prospective studies might look at the relationship among MRA arteriopathy, polysomnography, and hematology undertaken on the same day to further document the pathophysiology of cerebrovascular disease in SCD.

Early correction of hemoglobin oxygen desaturation, for example with hydroxyurea,^32^ adenotonsillectomy,^33^ or auto-adjusting continuous positive airway pressure,^34^ may reduce the risk of CNS events in children. Strategies, justifiable and feasible in young children, to improve hemoglobin oxygen saturation should be explored with the goal of preventing or reversing the vasculopathy and reducing the risk of stroke in this population.

## GLOSSARY

ANOVA: analysis of variance
ICA: internal carotid artery
IQR: interquartile range
MCA: middle cerebral artery
MR: magnetic resonance
MRA: magnetic resonance angiography
NOD: nocturnal oxygen desaturation
OSA: obstructive sleep apnea
SCD: sickle cell disease
STOP: Stroke Prevention Trial in Sickle Cell Anemia
TCD: transcranial Doppler
TE: echo time
TR: repetition time.
